# CSA and CSB play a role in the response to DNA breaks

**DOI:** 10.18632/oncotarget.24342

**Published:** 2018-01-29

**Authors:** Barbara Pascucci, Alessandra Fragale, Veronica Marabitti, Giuseppe Leuzzi, Angelo Salvatore Calcagnile, Eleonora Parlanti, Annapaola Franchitto, Eugenia Dogliotti, Mariarosaria D’Errico

**Affiliations:** ^1^ Institute of Cristallography, Consiglio Nazionale delle Ricerche, Roma, Italy; ^2^ Section of Tumor Immunology, Department of Oncology and Molecular Medicine, Istituto Superiore di Sanità, Roma, Italy; ^3^ Section of Mechanisms, Biomarkers and Models, Department of Environment and Health, Istituto Superiore di Sanità, Roma, Italy; ^4^ Department of Genetics and Development, Columbia University Irving Medical Center, New York, NY, USA

**Keywords:** Cockayne syndrome, DNA damage, DNA repair, γ-H2AX

## Abstract

CS proteins have been involved in the repair of a wide variety of DNA lesions. Here, we analyse the role of CS proteins in DNA break repair by studying histone H2AX phosphorylation in different cell cycle phases and DNA break repair by comet assay in CS-A and CS-B primary and transformed cells. Following methyl methane sulphate treatment a significant accumulation of unrepaired single strand breaks was detected in CS cells as compared to normal cells, leading to accumulation of double strand breaks in S and G2 phases. A delay in DSBs repair and accumulation in S and G2 phases were also observed following IR exposure. These data confirm the role of CSB in the suppression of NHEJ in S and G2 phase cells and extend this function to CSA. However, the repair kinetics of double strand breaks showed unique features for CS-A and CS-B cells suggesting that these proteins may act at different times along DNA break repair.

The involvement of CS proteins in the repair of DNA breaks may play an important role in the clinical features of CS patients.

## INTRODUCTION

Cockayne syndrome (CS) is a rare genetic progeroid disorder characterized by growth and development defects, severe cutaneous photosensitivity, cachectic dwarfism, progressive neurological dysfunction, and precocious aging. CS is most frequently due to mutations in either of two genes, *CSA* and *CSB*. Both gene products are involved in transcription-coupled repair (TCR), a sub-pathway of nucleotide excision repair (NER). Although CS-defective cells show hypersensitivity to UV light, impaired repair of bulky DNA lesions, delayed recovery of RNA synthesis after UV-damage, and enhanced apoptosis after transcription blockage (reviewed in [1]), patients with CS do not present increased cancer risk as expected in DNA repair defective syndromes. Furthermore, the characteristics of CS patients are hardly attributable to the NER impairment only, since some of them do not occur in XP-A patients, which show a complete NER deficiency [2]. CS cells present increased levels of intracellular reactive oxygen species (ROS), an intense glycolytic metabolism and mitochondria abnormalities [3–6]. A growing body of evidence indicate that, besides the typical NER lesions, CS cells are defective in the repair of a broad range of DNA damage that may account for the clinical symptoms of CS [7]. For instance, it is well established that, upon oxidative stress, CS-A and CS-B cells accumulate oxidatively induced DNA damage. In particular, CS cells are defective in the repair of 8-oxoguanine (8-OH-Gua), 5-hydroxycytosine (5-OHCyt) and cyclopurines [8–10]. CSB has been shown to participate to the repair of abasic sites [11] and in the coordination of interstrand crosslink (ICL) repair [12]. Moreover, it was reported that CS cells are hypersensitive to monofunctional alkylating agents, suggesting a defective repair of single strand breaks (SSBs) [11], and to agents known to induce double strand breaks (DSBs) such as ionizing radiation (IR) [13–15], camptothecin (CPT) [16] and etoposide [17]. Recently, CSB has been shown to repair DSBs by homologous recombination (HR) pathways at transcriptionally active sites [18, 19].

It is of note that most of the information available about the involvement of CS proteins in DNA break repair concerns CS-B cells. Since CSA and CSB play different roles, albeit interconnected in TC-NER, and since CS-A and CS-B patients present similar clinical features, it is important to improve our knowledge about the role of CSA in the maintenance of genomic stability.

Because a correct repair of DNA breaks is of potential great relevance for the clinical features of CS patients (defective DNA break repair is involved in neurodegeneration and neurogenesis) [20] here we analyse the role of CS proteins in single and double strand break repair (SSBR and DSBR) in primary and transformed CS-A and CS-B cells.

DNA break signalling and repair is analysed by the occurrence of histone H2AX phosphorylation (γ-H2AX) as a function of cell cycle and by alkaline and neutral single cell gel electrophoresis (SCGE). We provide the first evidence that CSA and CSB are involved in SSBR. Moreover, our data show a different repair kinetics of CSA and CSB cells suggestive of a differential role of these two proteins in the signalling/processing of DSBs.

## RESULTS

### Increased levels of DNA SSBs and a delay in their repair characterize CS fibroblasts after MMS-induced DNA damage

**Figure 1:**
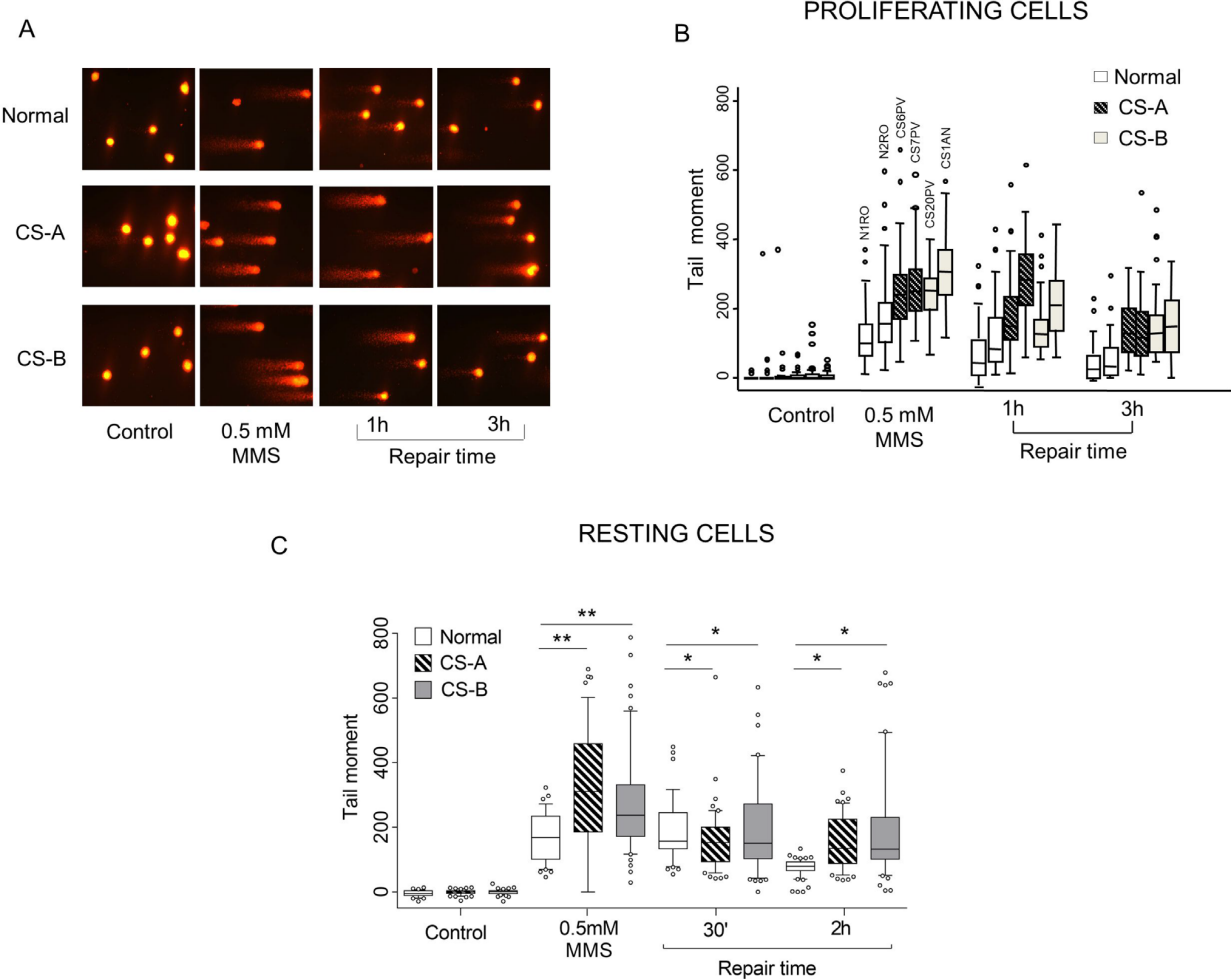
Evaluation of SSBs formation and repair in CS-A and CS-B cells after MMS exposure Normal and CS primary fibroblasts were exposed to 0.5 mM MMS for 30 min and repair kinetics was followed for different times. SSBs were measured by alkaline SCGE. Normal: N1RO and N2RO; CS-A: CS6PV and CS7PV; CS-B: CS20PV and CS1AN. (**A**) Microphotographs of normal, CS-A and CS-B cells, without treatment (control), immediately after treatment (0.5 mM) and after different repair times (1 h and 3 h). (**B**) Data from three independent experiments were reported as box plots. Each box encloses 50% of the data. The median of the distribution, the acceptable range and outliers are indicated. (**C**) Normal and CS primary fibroblasts synchronized in G1 phase were exposed to 0.5 mM MMS for 30 min and SSB were measured by alkaline SCGE and the repair kinetics was followed for different times (30 min and 2 h). Box plot shows data presented as tail moment. Box and whiskers represent 25–75 and 10–90 percentiles, respectively. The line represents the median value. Means of three independent experiments ± SE are reported; ^*^p < 0.01, ^**^p < 0.001 by nonparametric Wilcoxon ranksum test. Normal: N2RO; CS-A: CS6PV; CS-B: CS20PV.

A previous study indicates that CS cells are hypersensitive to monofunctional alkylating agents suggesting a defective repair of DNA breaks [11]. To investigate the role of CSA and CSB proteins in the response to alkylating agents-induced DNA damage, we monitored DNA breakage formation at single cell level by performing SCGE assay. To this aim, primary fibroblasts from two healthy subjects and two CS patients in each complementation group (CS-A and CS-B) were exposed to 0.5 mM of methyl methane sulphate (MMS) and the formation and repair of DNA SSBs as well as alkali labile sites (mainly abasic sites) were measured by SCGE. The number of SSBs at a given time point is the results of a dynamic process where breaks are formed and resealed at the same time. Although primary fibroblasts were affected by a significant inter-strain variability that is often associated with the use of cells derived from different subjects, a trend towards higher levels of DNA breaks immediately after treatment was noticed in CS cells as compared to cells from healthy subjects (Figure 1A and 1B). Hence, these results are suggestive of a defective DNA break repair mechanism in CS cells. This observation was confirmed by the significant delay in the repair of these BER intermediates observed at 3 h post-treatment (*p* < 0.005) (Figure 1A and 1B). Transformed CS fibroblasts complemented with the wild-type *CSA* or *CSB* genes confirmed the results obtained with primary fibroblasts (Supplementary Figure 1) reinforcing a role of CS proteins in the processing of DNA SSBs.

In order to determine whether the DNA breaks detected at late repair time (i.e. 3 h) are indeed a hallmark of defective SSBR, an alkaline comet assay in resting cells was performed. A significant enrichment of G1 phase cells (80% of the total cell population) was obtained by growing the cells to confluence followed by serum starvation for 48 hours (data not shown). Resting cells were treated with MMS (0.5 mM) for 30 min and allowed to repair for different repair times (30 min and 2 h) in drug-free medium. As shown in Figure 1C, similarly to what observed in proliferating cells, primary CS fibroblasts treated in G1 phase presented a significantly higher level of SSBs (*p* < 0.001) as compared to normal fibroblasts. Although after recovery the amount of SSBs appeared reduced in both CS-A and CS-B cells a significant accumulation of BER-intermediates was still detectable as compared to normal cells (*p* < 0.01). Therefore, our data in resting cells let us to conclude that SSBR is impaired in CS cells.

### The delay of CS cells in repairing MMS-induced DNA SSBs leads to increased levels of DSBs that are slowly repaired

**Figure 2:**
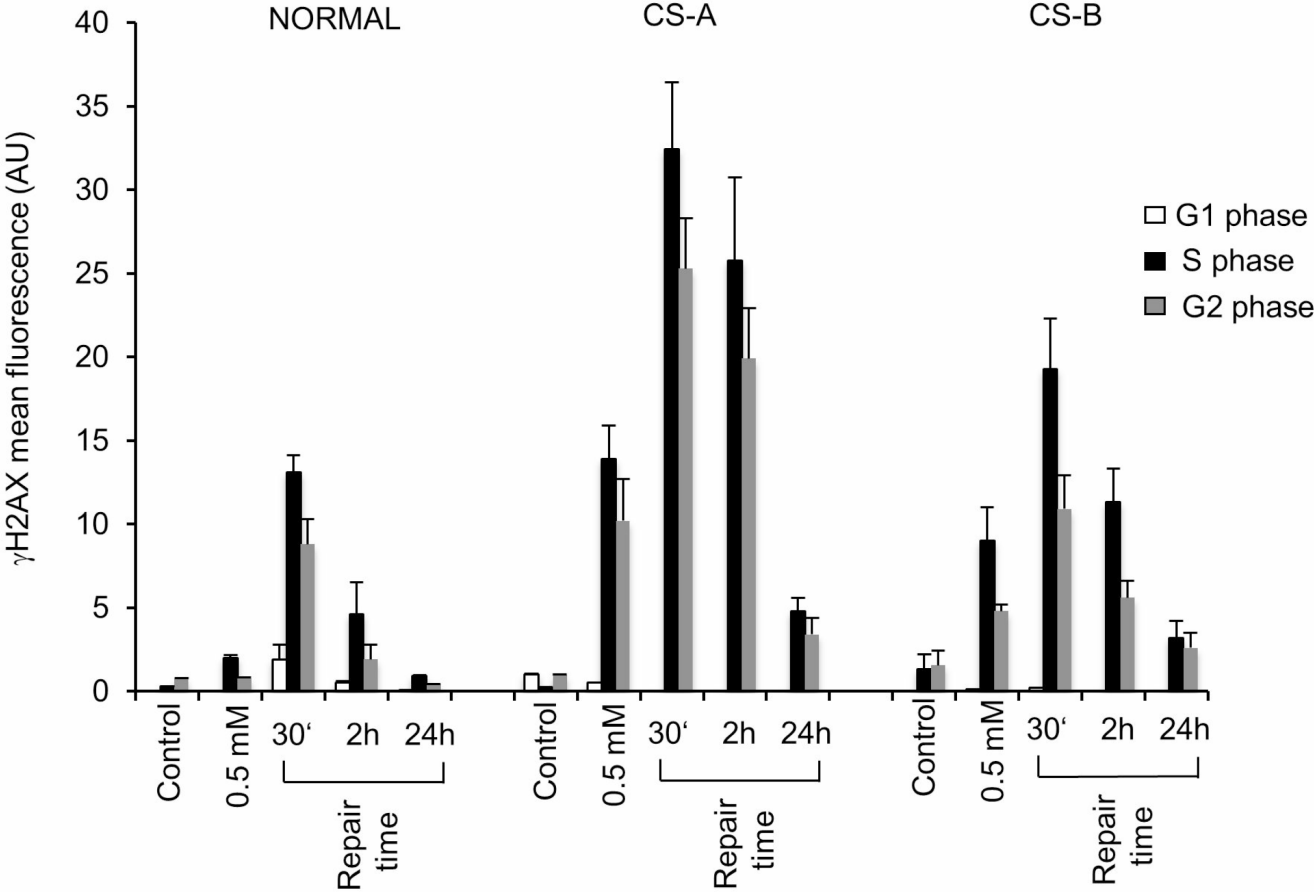
Flow cytometry analysis of histone H2AX phosphorylation in CS-A and CS-B cells in the different phases of the cell cycle after MMS treatment Normal, CS-A and CS-B primary fibroblasts were double stained with PI and an anti γ-H2AX antibody. The mean γ-H2AX fluorescence of G1-, S- and G2/M-phase selected cells of both untreated (control) and 0.5 mM MMS treated is shown. Normal: N2RO; CS-A: CS24PV; CS-B: CS20PV. Means of three independent experiments ± SE are reported. The mean fluorescence of γ-H2AX is expressed in arbitrary units (AU).

The accumulation of significant levels of SSBs after MMS treatment in G1-phase as observed in CS cells, is expected to generate DSBs in S-phase. H2AX phosphorylated form (referred as γ-H2AX) is considered an early sign of DNA damage induced by DNA replication fork stalling [21, 22], and a valuable marker of DSBs induction and processing [23, 24], although γ-H2AX foci signal the persistence also of SSB-containing DNA structures [25, 26]. Therefore, we measured DNA break accumulation using a specific anti-γH2AX antibody and its fluorescence intensity (FI) was evaluated by flow-cytometry analysis. Cells were also stained with propidium iodide (PI), thus giving the possibility to measure DSBs in large cell populations and concurrently to define the cell cycle phase in which DNA breaks are being detected [27–29]. Proliferating normal and CS mutant cells were treated with MMS for 30 min. As shown in Figure 2, high levels of γ-H2AX FI were measured in CS fibroblasts, particularly in CSA cells, immediately after MMS treatment (0.5 mM for 30 min), whereas very low levels were detected in normal fibroblasts. The percentage of cells into different cell cycle stages is reported for each cell line (Supplementary Table 1). Our analysis revealed that γ-H2AX FI was at most localized in S and G2-phase cells both in normal and CS cells in agreement with the occurrence of DSBs following replication of MMS-induced SSBs (Figure 2). However, the accumulation of DSBs in S and G2-phases, detected by γ-H2AX fluorescence signal, was significantly higher in CS cells than in normal cells at all repair times analysed (Supplementary Figure 2A and 2B). In particular, although some residual DNA damage was still present, the dephosphorylation of γ-H2AX was almost complete at 24 h in CS cells, whereas in normal fibroblasts this phenomenon was already observed at 2 h after MMS exposure (Figure 2). These results were confirmed using different strains of primary fibroblasts (Supplementary Figure 3). FACS analysis of S-phase gated cells (Supplementary Figure 4) allows to further appreciate the higher levels of γ-H2AX signal in CS cells, respect to normal cells, at both 30 min and 2 h post-treatment times.

The analysis in primary fibroblasts may be hampered by various factors including differences in the genetic background ([30, 31] and our unpublished observations) and therefore we further investigated the role of CSA and CSB in MMS-induced break repair by using also transformed cell lines. In particular, for CSA the study was conducted in the SV40-transformed CS-A cell line CS3BE and in its derivative CS3BE-wtCS-A as previously described ([6] and Lanzafame *et al*., manuscript in preparation). For CS-B we used the SV40-CS1AN transformed cell line and as its wild type counterpart the SV40-CS1AN-wtCS-B cell line stably transfected with wild-type *CSB* gene [11].

**Figure 3:**
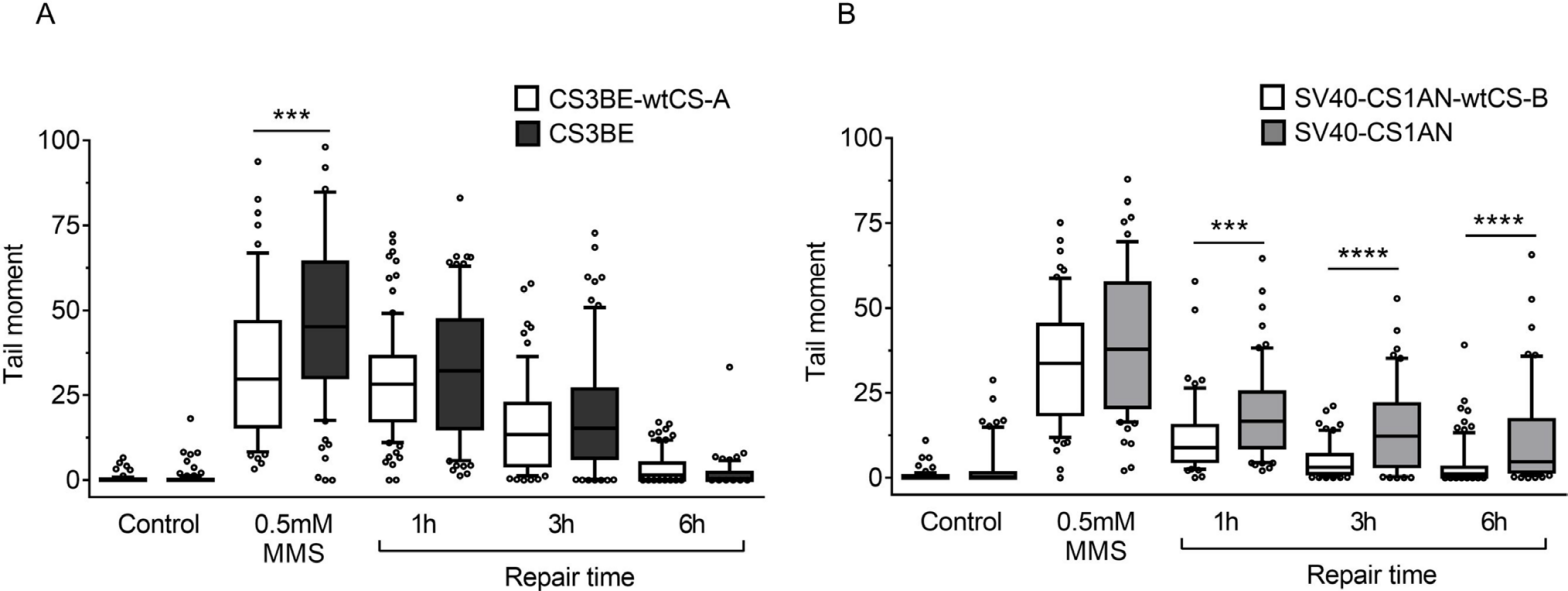
Evaluation of DSB formation and repair in CS-A and CS-B cells after MMS exposure Cells were treated with 0.5 mM MMS for 30 min and repair kinetics was followed for different times (1 h-6 h). DSBs were measured by neutral SCGE on CS-A (CS3BE) (**A**) and CS-B (SV40-CS1AN) cells (**B**) Box plot shows data presented as tail moment. Box and whiskers represent 25–75 and 10–90 percentiles, respectively. The line represents the median value. Means of three independent experiments are reported; ^**^p < 0.001, ^***^p < 0.0001, Kruskal-Wallis test multicomparison Anova.

A neutral comet assay was performed in order to monitor specifically DSBs induction and repair. Normal and defective transformed cell lines were treated with 0.5 mM MMS for 30 min. Increased comet tail moment values were reported in CS3BE (CS-A) cells immediately after treatment as compared to the isogenic wild-type cell line (*p* < 0.001), whereas at longer repair time the values were indistinguishable between normal and defective cells (Figure 3A). A different behaviour was observed in CS-B cells (Figure 3B). Indeed, SV40-CS1AN cells showed a slight increase in the comet tail moment after MMS treatment, but a strong delay in DSBR at all the repair times analysed (*p* < 0.001 for 1 h repair and *p* < 0.0001 for later repair times) (Figure 3B). These data support the hypothesis that CSA and CSB are involved in DSBR, and suggest their intervention at different times along the repair process of DSBs. It is of note the dissociation between the kinetics of H2AX phosphorylation that is identical in CS-A and CS-B cells and that of DSBR that distinguishes CS-A from CS-B cells (see Discussion).

All together, these data indicate that, following MMS treatment, CS-A and CS-B cells are defective in SSBR leading to higher levels of H2AX phosphorylation when unrepaired SSBs encounter replication fork. In addition, when the physical repair of DSBs is monitored by the neutral comet assay a defect in DSBR is detected in CS-B but not in CS-A cells that had a role only at early time after MMS treatment.

### CSA and CSB are involved in the repair of double strand breaks

In order to better characterize the role of CS proteins in DSBR, we treated primary CS-A or CS-B fibroblasts with ionizing radiation (IR). Although IR are known to induce a broad range of DNA lesions, γ-H2AX FI in G0/G1-phase represents the signalling triggered by DSBs and is used to monitor their disappearance [32].

**Figure 4:**
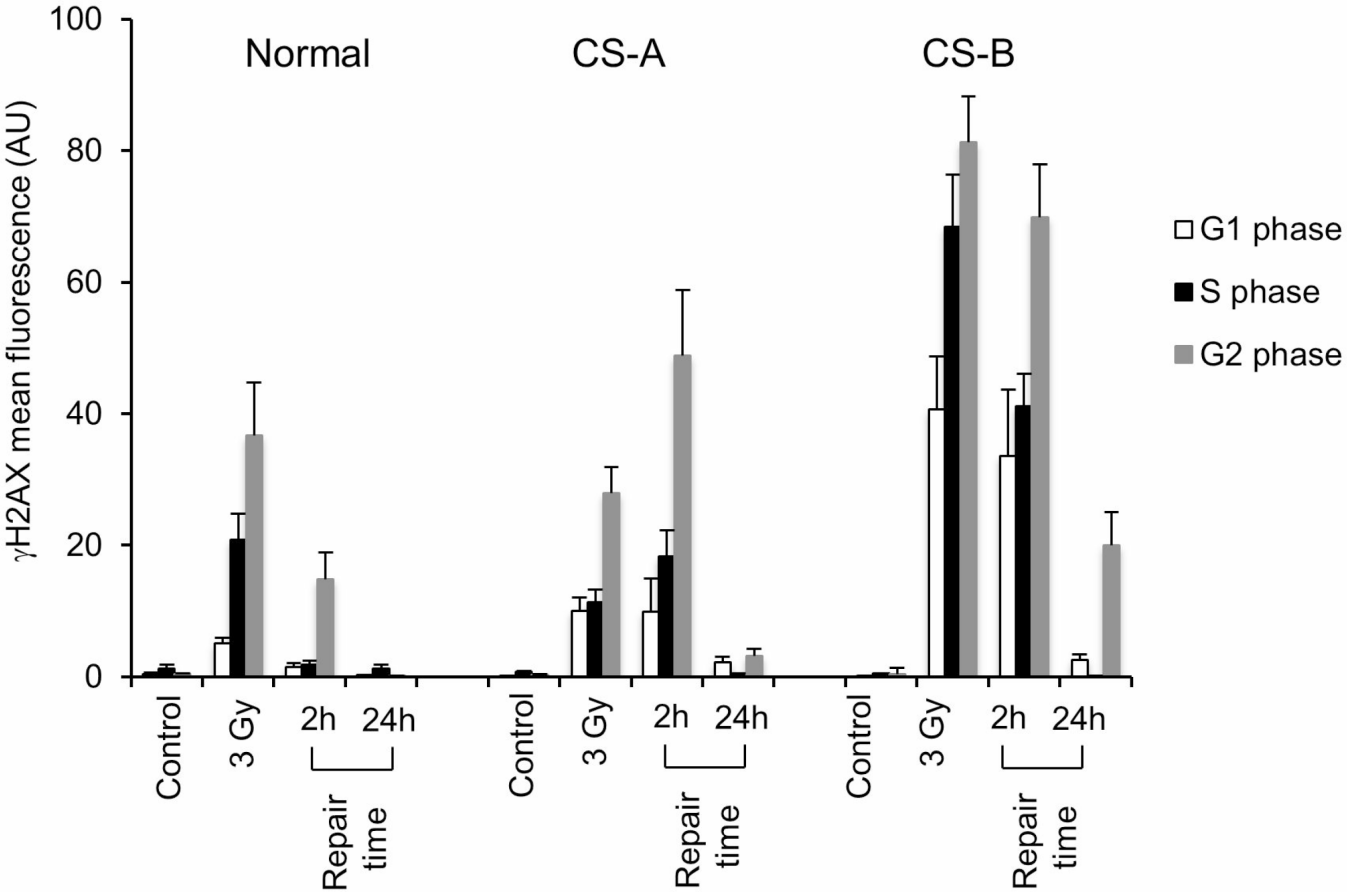
Flow cytometry analysis of histone H2AX phosphorylation on CS-A and CS-B cells in the different phases of the cell cycle after IR Normal, CS-A and CS-B primary fibroblasts were double stained with PI and an anti γ-H2AX antibody. The mean γ-H2AX fluorescence of G1-, S- and G2-phase selected cells of both untreated (control) and 3 Gy ionizing radiation treated is shown. Normal: N2RO; CS-A: CS24PV; CS-B: CS20PV. Means of three independent experiments ± SE are reported. The mean fluorescence of γ-H2AX is expressed in arbitrary units (AU).

Hence, proliferating normal or CS mutant cells were treated with 3 Gy, and allowed to recover for the indicated times, then γ-H2AX FI was evaluated by flow-cytometry as above. As shown in Figure 4, accumulation of cells with high levels of γ-H2AX FI, as detected by cytofluorimetric analysis, was observed in both CS-A and CS-B cells primary fibroblasts as compared to normal fibroblasts at 2 h repair time (*p* < 0.01). This phenomenon was more remarkable in CS-B cells that showed a higher level of γ-H2AX FI already after exposure to 3 Gy of IR when compared to CS-A cells (Figure 4). Complete dephosphorylation of H2AX was detected at 24 h post-treatment time in primary fibroblasts with some residual damage still present in CS-B cells (Figure 4). Similar results were obtained in transformed fibroblasts (Supplementary Figure 5A–5D) and by using different strains of primary fibroblasts (Supplementary Figure 6). Immunofluorescence analysis confirmed that the majority of γ-H2AX foci colocalized with 53BP1 either in transformed cells (Supplementary Figure 5E and 5F) or in primary fibroblasts (Supplementary Figure 7) as expected when NHEJ-mediated DSB repair is occurring [18]. The analysis of distribution of γ-H2AX positive cells as a function of the cell cycle by double staining for γ-H2AX and DNA content, revealed that the γ-H2AX fluorescence signal was at most localized in S and G2 cells, both in normal and CS cells (Figure 4), as also observed after MMS-treatment (Figure 2), but also in G1 cells (Figure 4). As expected, this category of breaks is missing in the profile of MMS-treated cells (Figure 2) where DSBs arise from replication of unrepaired SSBs. Moreover, the accumulation of DSBs in all cell cycle phases was significantly higher in CS cells than in normal cells after 2 h of repair time ( Supplementary Figure 8). At 24 h of repair time the dephosphorylation of γ-H2AX was almost complete either in CS and normal cells (Supplementary Figure 8).

**Figure 5:**
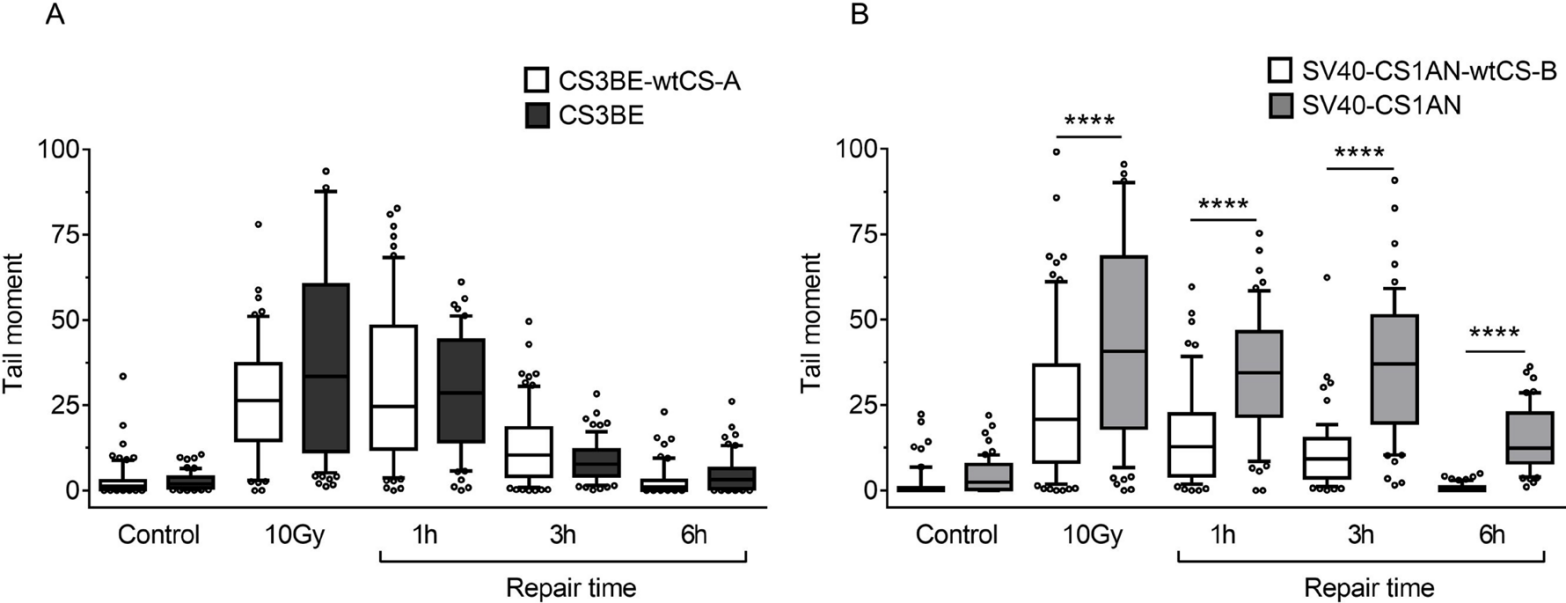
Evaluation of DSB formation and repair in CS-A and CS-B cells after exposure to IR Cells were treated with 10 Gy of ionizing radiation and repair kinetics was followed for different times (1 h-6 h). DSBs were measured by neutral SCGE on CS-A (CS3BE) (**A**) and CS-B (SV40-CS1AN) (**B**) transformed fibroblasts, and their isogenic derivatives expressing the wild-type genes (CS3BE-wtCS-A and SV40-CS1AN-wtCS-B). Box plot shows data presented as tail moment. Box and whiskers represent 25–75 and 10–90 percentiles, respectively. The line represents the median value. Means of three independent experiments are reported; ^**^p < 0.001, ^***^p < 0.0001, Kruskal-Wallis test multicomparison Anova.

In order to assign the defect in DSBR unequivocally to CS proteins, the isogenic CS-A and CS-B wild-type and defective transformed cell lines were used to perform a neutral comet assay after IR exposure (10 Gy). As shown in Figure 5A, in the CS3BE cells (CS-A) an increase (although not statistically significant) in the comet tail moment was visible immediately after treatment as compared to the wild type cells. By contrast, no differences between the two cell lines, during different repair times were detected (Figure 5A). A different behaviour was observed in the CS-B cells. In these cells the comet tail moment immediately after treatment was higher (*p* < 0.0001) than that observed in the normal counterpart (Figure 5B) but, in addition, the CS-B cells presented a strong delay (*p* < 0.0001) in DSBR at all the repair times analysed (Figure 5B). This difference was also observed when MMS was used (Figure 3), thus supporting the hypothesis of a different role played by CSA and CSB in DSBR.

The dissociation between the kinetics of H2AX phosphorylation and that of DSBR observed previously after MMS treatment was observed also after IR treatment (see Discussion).

## DISCUSSION

In this study, we provide evidence that CS proteins are involved in the recognition, signalling and processing of SSBs as well as DSBs. Defects in the cellular response to DNA strand breaks may be relevant for the clinical features of CS patients.

SSBR is generally considered a specialized sub-pathway of BER, since it often engages proteins dedicated to BER. SSBR copes with normal and abnormal strand breaks that arise either from reactions with DNA-damaging agents (endogenous or exogenous) or as intermediates in certain enzymatic events. In this study, we investigated whether CSA and CSB are involved in SSBR. We show that MMS-treated CS cells are more prone to breakage and present a delay in SSBR, as unequivocally confirmed in resting primary cells. If cells enter into the S phase with a load of unrepaired SSBs, the likelihood of DSBs formation is expected to increase. Indeed, after MMS treatment, higher and persisting levels of H2AX phosphorylation were observed in CS cells in S and G2 phases as compared to normal cells. All these data provide the first evidence of involvement of both CSA and CSB in SSBR, and may explain the cell hypersensitivity to the killing effects of MMS previously reported [11]. It is well known that CSB interacts with several BER proteins (e.g. PARP1, APE1 and OGG1) [11, 33, 34], stimulating their activity (e.g. Neil1, APE1) [9, 11, 15, 35] and affecting their transcription (as in the case of OGG1) [36]. Interestingly, it has been shown that the accumulation of DSBs in ATM-deficient cells is due to a defect in the signalling of unrepaired SSBs, leading to DSBs as a result of the replication of SSB-containing DNA [37]. Furthermore, it has been reported that an alkylating agent, such as MNNG, can cause excess of DNA strand breaks that lead to poly(ADP-ribose)polymerase-1 (PARP-1) over-activation and cell death *via* ROS production [38]. Whether the delay in MMS-induced SSBR of CS-deficient cells is due to lower N-methylpurine DNA glycosylase (MPG), APE1 activity or to the participation of CS proteins in SSBs signalling, awaits to be investigated.

In this paper, we show that CS cells are also defective in DSBs signalling and repair. Increased and persisting high levels of H2AX phosphorylation following IR exposure, as well as the co-localization of γ-H2AX foci with 53BP1 (a crucial component of DNA DSB signalling and repair in mammalian cells), indicate a clear role of CS proteins in the signalling/processing of DSBs. Once a DSB occurs within a cell, there is a rapid, concerted signalling cascade to process the damage and to prevent adverse consequences that could impact cellular function and survival. Recently, CSB has been shown to promote the homologous recombination (HR) pathway at transcriptionally active sites, and to repress the non-homologous end-joining (NHEJ) in S and G2-phase cells by its ATPase activity [18, 19, 29, 35]. Accordingly, our data revealed that in CS-A and CS-B cells, the γ-H2AX fluorescence is mostly localized in S and G2 cells. It is well known that CSB has an ATP-dependent chromatin remodelling activity *in vitro* [39, 40] that may influence the choice of the DSB repair pathway, especially in S and G2 phases of the cell cycle during which either the HR or NHEJ pathways could be chosen [41–45].

It has been questioned whether the phosphorylation of H2AX may identify also DNA damage-containing structures other than DSBs. In the last decade, many papers highlighted the role of the biological scenario (i.e. the cell type, the chromatin structure and the presence of other remodelling complexes) on the activities that H2AX phosphorylation may signal/trigger [25, 46, 47]. Indeed, in this study a discrepancy is observed between the kinetics of H2AX phosphorylation/dephosphorylation and DSBR as detected by the neutral comet assay. In particular, while both CS-A and CS-B cells show persisting high levels of H2AX phosphorylation after MMS or IR exposure when compared to normal cells (Figures 2 and 4), the DSBs rejoining rate is normal in the CS-A cells and delayed in the CS-B cells (Figures 3 and 5). We may speculate that while γ-H2AX registers cellular metabolic activities initiated to facilitate and optimize DSBR [24], activities that are defective in both CSA and CSB, the neutral comet assay measures physical DSBR that is defective in CSB only. In agreement with our data, Wei and colleagues [19] showed that *CSB* but not *CSA* silencing affects HR factor recruitment to transcriptionally active damage sites. It should also be noted that high IR doses (above 5 Gy) are required to measure DSBs by physical methods such as the neutral comet assay, while H2AX phosphorylation is measured at doses well below 5 Gy [48]. We cannot exclude that different pathways are triggered at low versus high IR doses.

In this paper, we have noticed a different DSBR rate between CS-A and CS-B cells suggesting a different role of CS proteins in the recognition and repair of DNA breaks. As in the case of UV damage, these two proteins may act in different time windows during the repair pathway [49, 50].

The accumulation of persistent γ-H2AX foci with age [51, 52] is a hallmark of the aging process [53]. DSBs are the main lesions responsible for genomic instability and cell death. Our findings of defective DSBR in CS cells is compatible with their susceptibility to spontaneous and stress-induced apoptosis (reviewed in [54]). In a previous study, we have shown that CS cells are defective in mitophagy and the stimulation of this process by Parkin leads to an anti-apoptotic effect [6]. It has been shown that replication stress-derived nuclear DNA damage may arise as a consequence of mitochondrial dysfunction [55]. We may envisage a scenario where both altered mitochondria [3–6, 56] and defective DNA break repair (this paper, [18, 19]) contribute to the increased load of DSBs in CS cells, thus accounting for increased apoptosis and the molecular and clinical features of this syndrome.

## MATERIALS AND METHODS

### Cell culture and treatment conditions

Normal (N2RO and N3RO), CS-A (CS6PV and CS24PV) and CS-B (CS1PV and CS23PV) primary fibroblasts and SV40-transformed cell lines ([6, 9] and Lanzafame *et al*., manuscript in preparation) were established and cultured as previously described [57]. The CS1ANwtCS-B (HACS1AN) and CS1AN cell line was a gift of Dr. Hoeijmakers JH, Rotterdam, NL.

Treatments with MMS (0.5 mM) were performed in DMEM supplemented with 20 mM Hepes for 30 min. Cells were treated with X-rays (3 and 10 Gy) (Radgil, Gilardoni, Milan, Italy).

Cells were synchronized in G1 and S phases by growth to confluence and incubated in low serum (0.5% FCS) containing medium for 48 h.

### Single cell gel electrophoresis (SCGE)

Primary and SV-40 transformed fibroblasts were treated with MMS or X-rays, and then subjected to the alkaline comet assay as previously described [58] and at least 100 comets per each experimental point were analysed using an Image Analysis Software (IAS, Delta Sistemi, Rome, Italy). DNA break levels were compared by nonparametric Wilcoxon ranksum test. The level of heterogeneity between mean values in different donor groups was assessed by a one-way analysis of variance. Because of differences in the morphology and migration response to damage in transformed and primary fibroblasts, the tail moments cannot be compared between these different cell types [59].

DNA double strand breaks formation was also evaluated in non-denaturing conditions by neutral comet assay (as described in [60]). Briefly, dust-free frosted-end microscope slides were kept in methanol overnight to remove fatty residues. Slides were then dipped into molten Normal Melting Point (NMP) agarose at 1% and left to dry. Cell pellets were resuspended in PBS and kept on ice to inhibit DNA repair. Cell suspensions were rapidly mixed with Low Melting Point (LMP) agarose at 0.5% kept at 37° C and an aliquot was pipetted onto agarose-covered surface of the slide. Agarose embedded cells were lysed by submerging slides in lysis solution (30 mM EDTA, 0.1% SDS, 0.5 mg/ml Proteinase K) and incubated 1 h in the dark. After lysis, slides were washed in TBE 1X running buffer (Tris 90 mM; boric acid 90 mM; EDTA 4 mM). Electrophoresis was performed for 25 min in TBE 1X buffer at 0.6 V/cm. Slides were subsequently washed in distilled water and finally dehydrated in ice cold methanol. Nuclei were stained with GelRed (1:1000) and visualized with a fluorescence microscope (Zeiss), using a 20X objective, connected to a CCD camera for image acquisition. At least 300 comets per each experimental point were analysed using TriTek Comet Score software and data from tail moments processed using Prism software. Apoptotic cells (smaller Comet head and extremely larger Comet tail) were excluded from the analysis to avoid artificial enhancement of the tail moment. DNA breaks were compared by Kruskal-Wallis test multicomparison Anova.

### Immunostaining and flow-cytometry

A flow cytometry based method was used to analyse γ-H2AX [28] with minor modifications. Briefly, cells were collected, fixed in cold 70% ethanol, washed, rehydrated and stained with anti-phosho-histone H2AX (Ser139) FITC conjugated mAb (Millipore,16–202A), in TBS containing 4% FBS and 0.1% Triton X-100. Cells were treated with RNase A and DNA was stained with PI (Sigma-Aldrich). A minimum of 20.000 stained cells were acquired on a FACS Calibur (Becton Dickinson) at low speed.

### Immunofluorescence staining for γ-H2AX and 53BP1

Cells were grown in 35-mm coverslips and harvested at the indicated times after treatment. For 53BP1 IF, after further washing with PBS, cells were fixed with 4% PFA at RT for 10 min. Cells were subsequently permeabilized with 0.4% Triton-X100. Staining with mouse polyclonal anti-53BP1 (Calbiochem), rabbit monoclonal anti-γ-H2AX (Ser 139; Millipore, Billerica, MA) in a 1%BSA/0,1% saponin in PBS solution, was carried out for 1 h at RT. After extensive washing with PBS, specie-specific fluorophore-conjugated antibodies (Invitrogen) were applied for 1 h at RT followed by counterstaining with 0.5 mg/ml DAPI. Secondary antibodies were used at 1:200 dilution. Coverslips were analysed using a Leica DRMB fluorescence microscope equipped with a charge coupled device camera. The images were processed using the IAS 2000 Delta System software (Adobe, San José, CA).

### Note added in proof

When the paper was under revision, the study by Batenburg *et al*, showing that ATM and CDK2 control the chromatin remodeling activity of CSB in the regulation of DSB repair pathway choice, was published in Nature Communications [61].

## SUPPLEMENTARY MATERIALS FIGURES AND TABLES


