# Supplementary material for: CSA and CSB play a role in the response to DNA breaks
Source: Oncotarget. 2018 Jan 29;9(14):11581–91. doi: 10.18632/oncotarget.24342 (PMC5837770; doi:10.18632/oncotarget.24342)
Supplement: Supplementary file 1 [file oncotarget-09-11581-s001.pdf]

CSA and CSB play a role in the response to DNA breaks

SUPPLEMENTARY MATERIALS

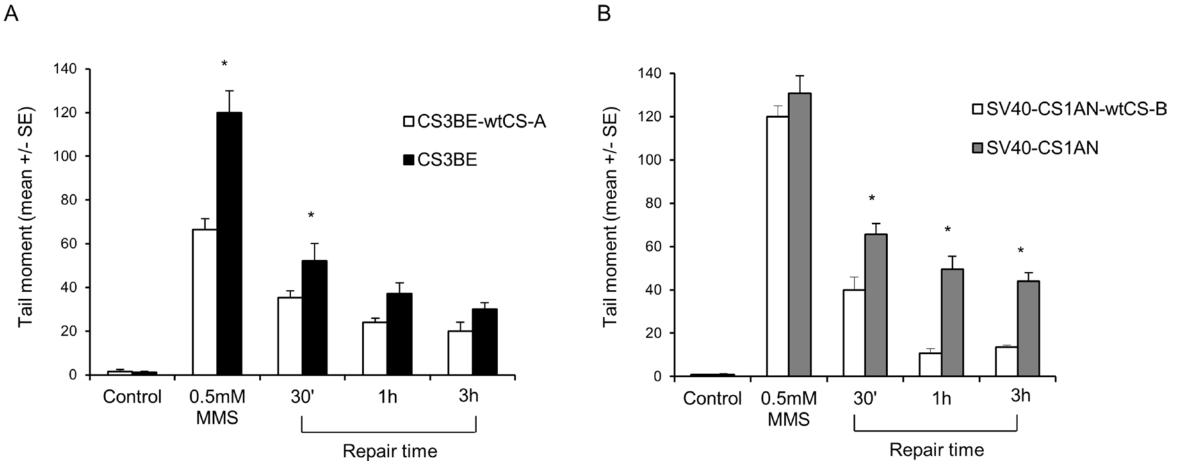

Supplementary Figure 1: Evaluation of SSBs formation and repair in CS-A and CS-B cells after MMS exposure. Normal and CS transformed fibroblasts were exposed to 0.5 mM MMS for 30 min and repair kinetics was followed for different times (0.5 h–3 h). SSBs were measured by alkaline SCGE on CS-A (CS3BE) (**A**) and CS-B (SV40-CS1AN) (**B**) transformed fibroblasts, and their isogenic derivatives expressing the wild-type genes (CS3BE-wtCS-A and SV40-CS1ANwtCS-B). Bar graphs show data presented as mean tail moment ± SE. $^{*}p < 0.01$, by nonparametric Wilcoxon ranksum test.

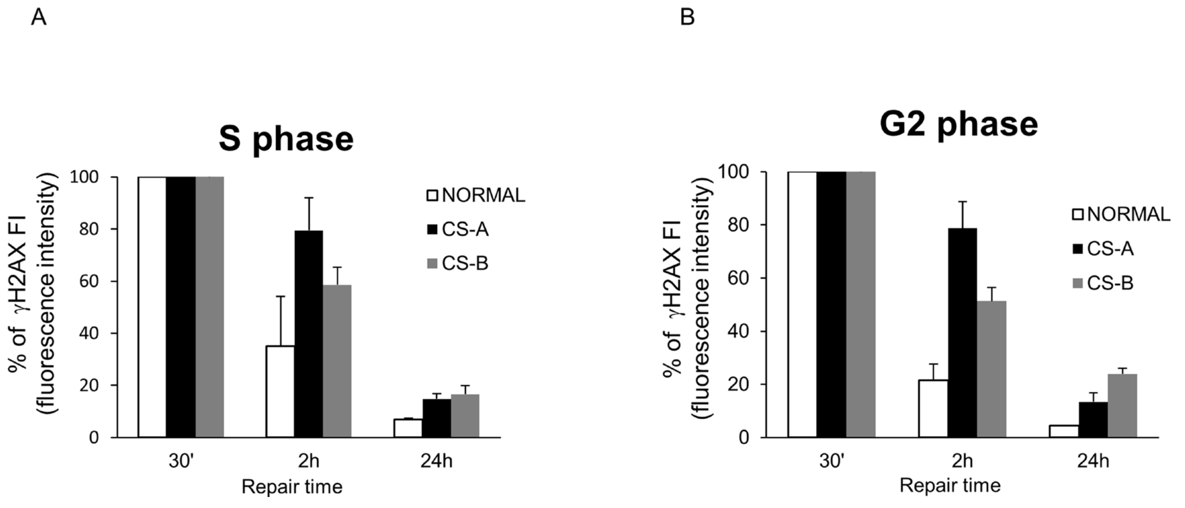

Supplementary Figure 2: Flow cytometry analysis of histone H2AX phosphorylation in CS-A and CS-B primary fibroblasts, in the different phases of the cell cycle after MMS treatment. In (**A**) was reported the percentage of γ-H2AX fluorescence intensity in CS-A and CS-B cells in S phase, in (**B**) data collected in G2 phase, after MMS treatment.

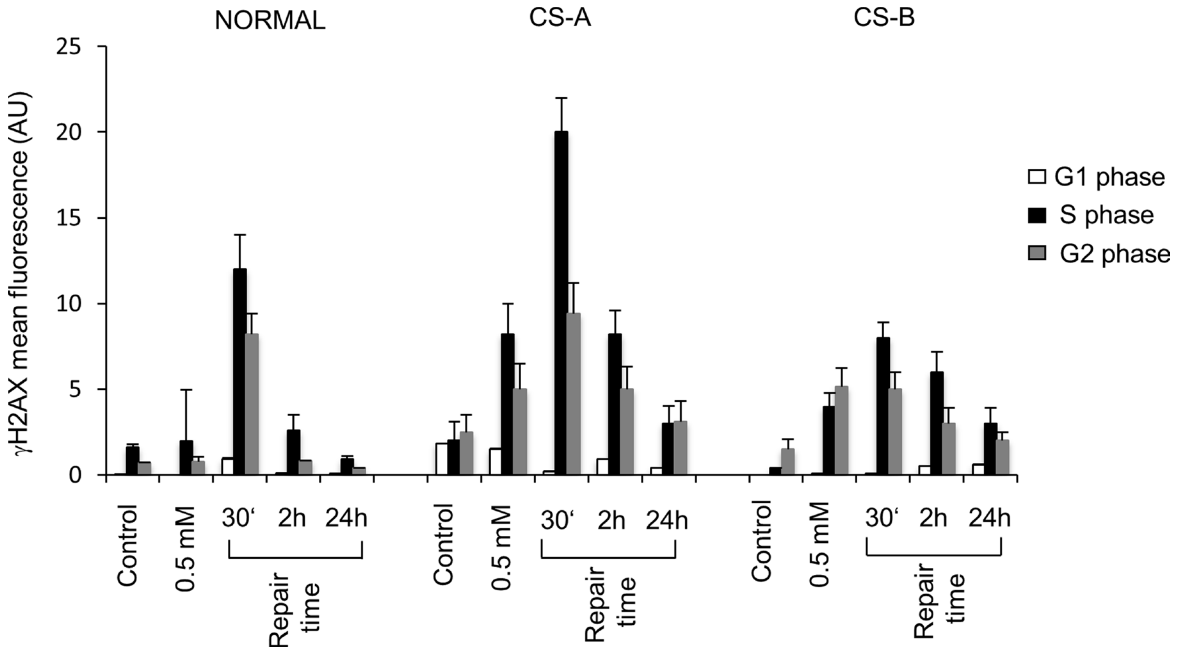

Supplementary Figure 3: Flow cytometry analysis of histone H2AX phosphorylation in CS-A and CS-B cells in the different phases of the cell cycle after MMS treatment. Normal, CS-A and CS-B primary fibroblasts were double stained with PI and an anti γ-H2AX antibody. The mean γ-H2AX fluorescence of G1-, S- and G2/M-phase selected cells of both untreated (control) and 0.5 mM MMS treated is shown. Normal: N1RO; CS-A: CS6PV; CS-B: CS23PV. Means of three independent experiments ± SE are reported. The mean fluorescence of γ-H2AX is expressed in arbitrary units (AU).

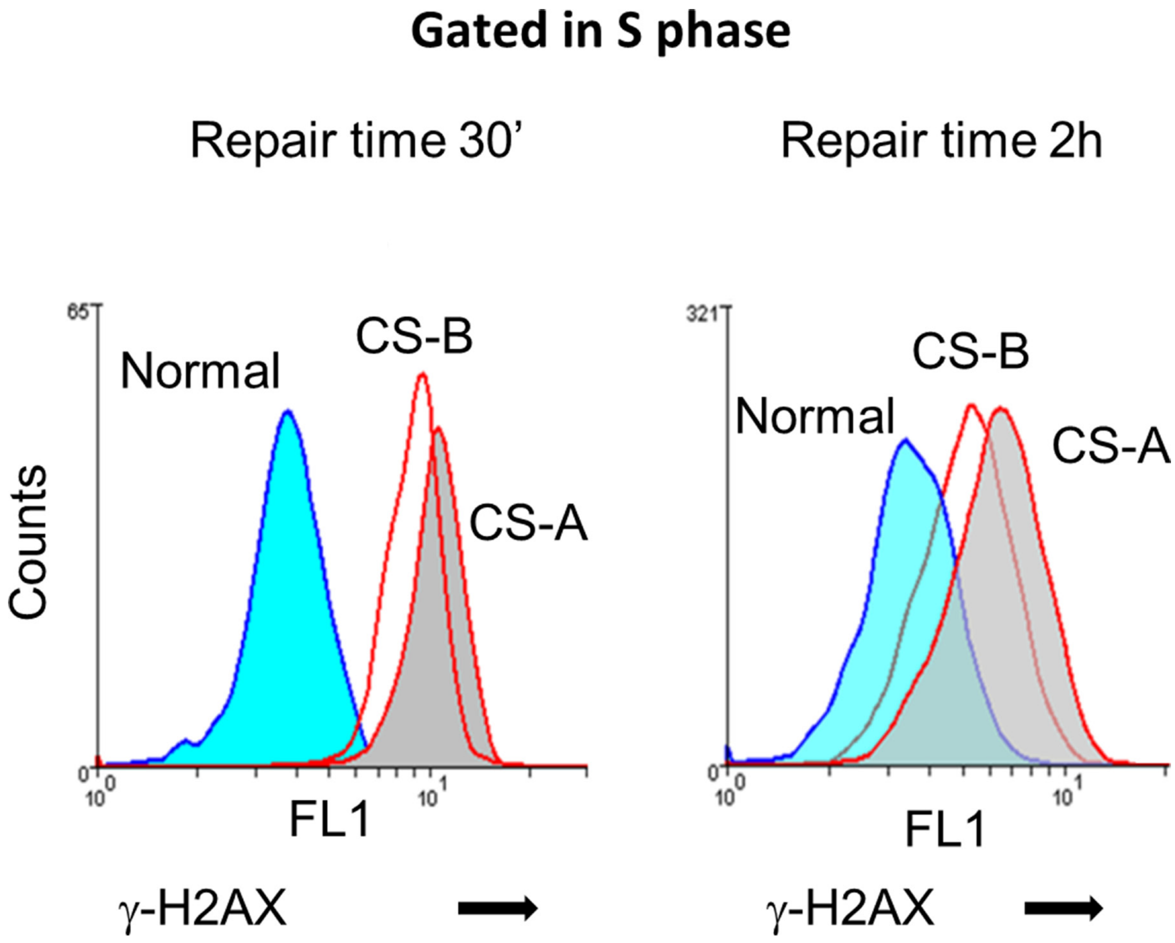

Supplementary Figure 4: γ-H2AX fluorescence intensity of S-phase gated cells was analyzed after different repair times (30 min and 2 h). Normal: N2RO; CS-A: CS24PV; CS-B: CS20PV. Histograms were representative of one of three experiment performed. Results were confirmed with other primary fibroblasts from normal and defective cells.

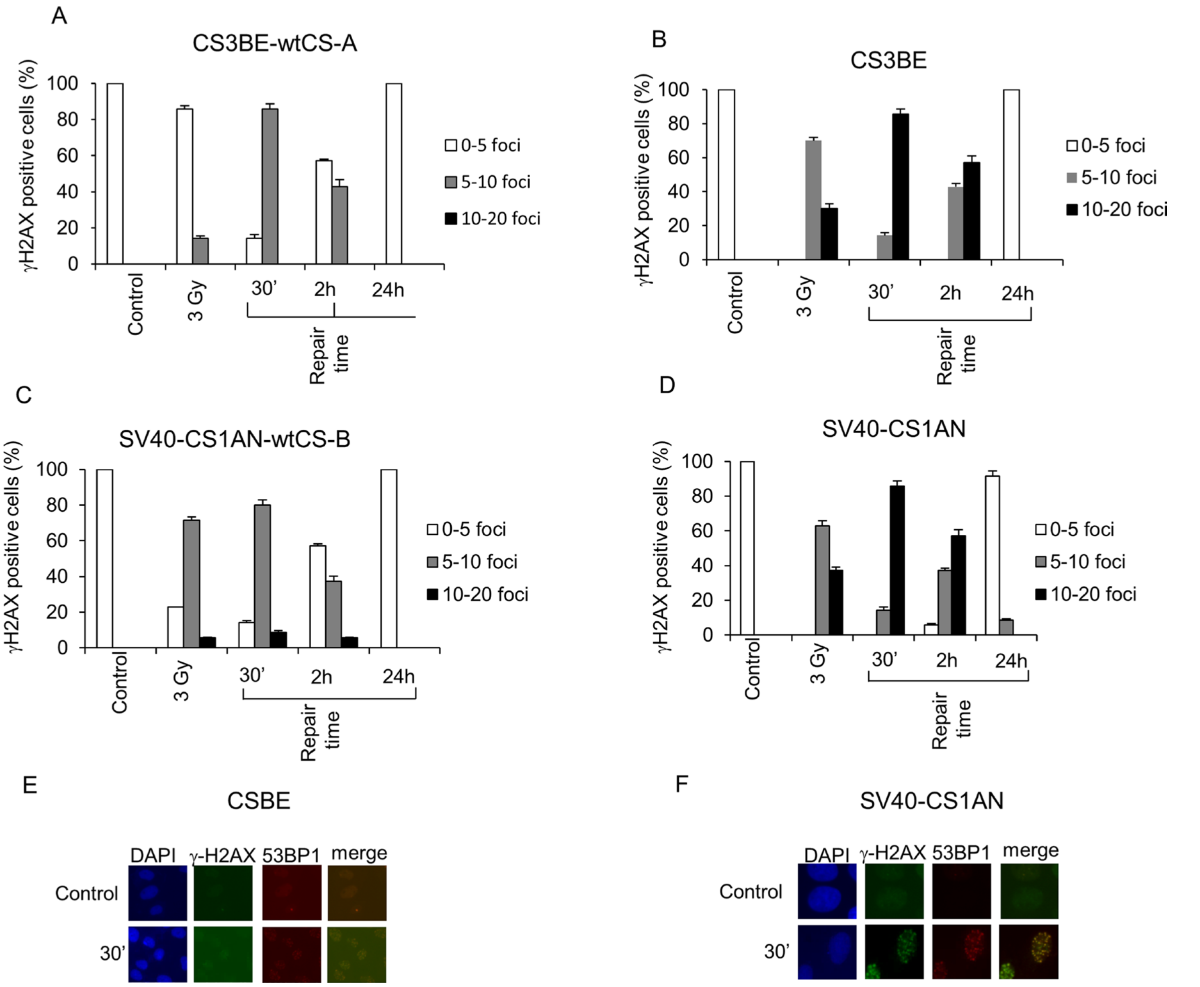

Supplementary Figure 5: Immunofluorescence analysis of γ-H2AX foci in CS transformed fibroblasts after IR. Cells were treated with 3 Gy of ionizing radiation and repair kinetics was followed for different times (30 min-24 h). (**A**) Isogenic transformed fibroblasts expressing the wild-type CS-A gene (CS3BE-wtCS-A), (**B**) CS-A transformed defective cells (CS3BE), (**C**) isogenic transformed fibroblasts expressing the wild-type CS-B gene (SV40-CS1AN-wtCS-B) and (**D**) CS-B transformed defective cells (SV40-CS1AN); (**E**) and (**F**) spatial distribution of γ-H2AX (green) and 53BP1 (red) foci are shown in representative nuclei of CS3BE (CS-A) and SV40-CS1AN (CS-B) transformed cells under high magnification. Images were merged to determine colocalization.

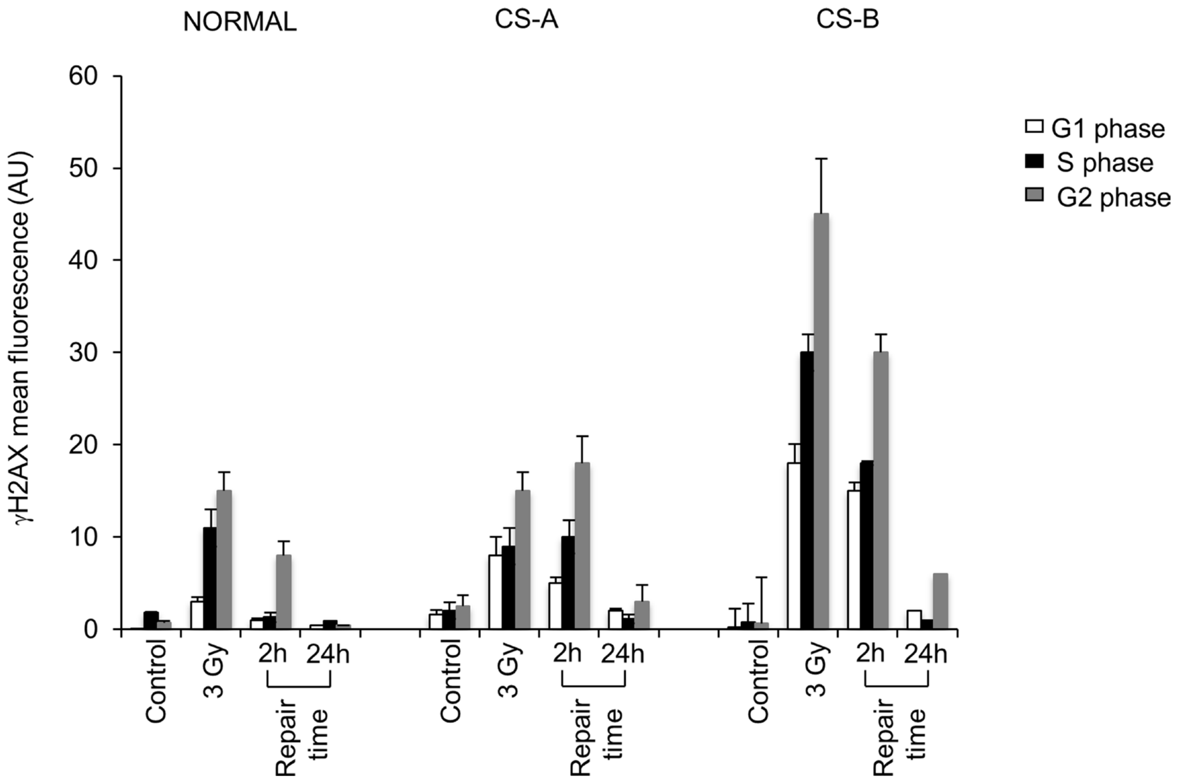

Supplementary Figure 6: Flow cytometry analysis of histone H2AX phosphorylation on CS-A and CS-B cells in the different phases of the cell cycle after IR. Normal, CS-A and CS-B primary fibroblasts were double stained with PI and an anti γ-H2AX antibody. The mean γ-H2AX fluorescence of G1-, S- and G2-phase selected cells of both untreated (control) and 3 Gy ionizing radiation treated is shown. Normal: N2RO; CS-A: CS6PV; CS-B: CS23PV. Means of three independent experiments ± SE are reported. The mean fluorescence of γ-H2AX is expressed in arbitrary units (AU).

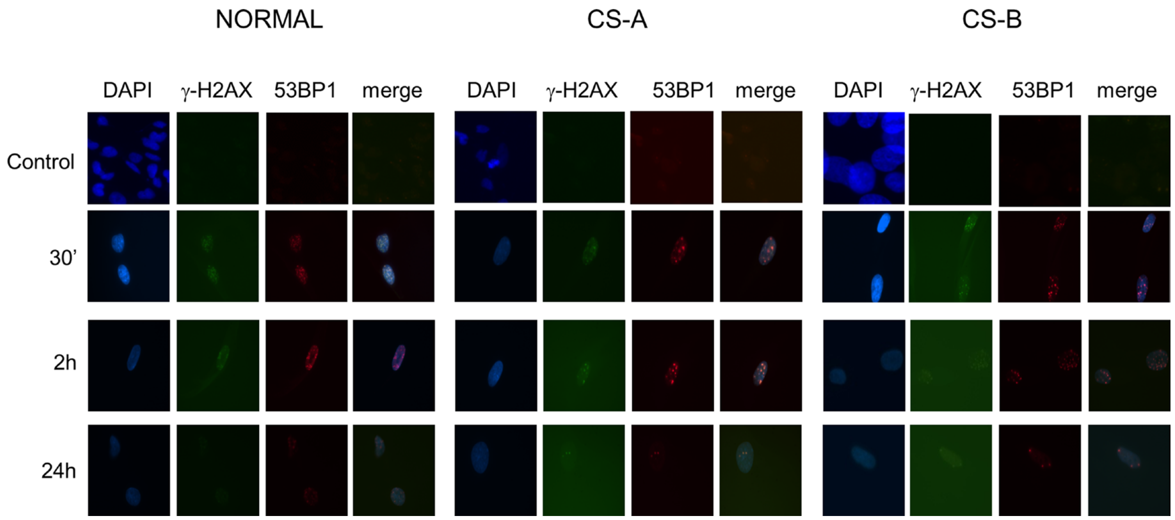

Supplementary Figure 7: Immunofluorescence analysis of colocalization of γ-H2AX foci with 53BP1 in CS-A and CS-B cells after IR. The spatial distribution of γ-H2AX (green) and 53BP1 (red) foci are shown in representative nuclei of normal, CS-A and CS-B cells under high magnification. Images were merged to determine colocalization. Normal: N2RO; CS-A: CS24PV; CS-B: CS20PV.

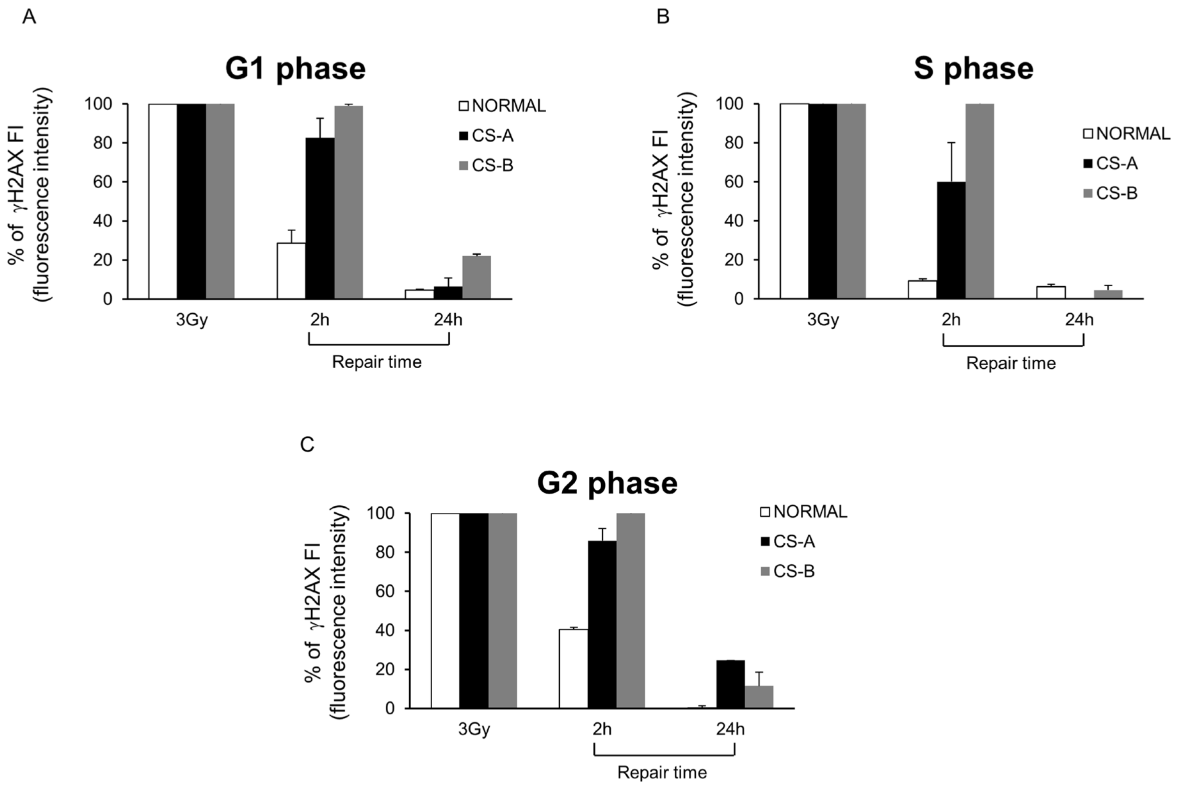

Supplementary Figure 8: Flow cytometry analysis of histone H2AX phosphorylation in CS-A and CS-B cells in the different phases of the cell cycle after IR treatment. In (**A**) is reported the percentage of γ-H2AX fluorescence intensity in CS-A and CS-B cells in G1, in (**B**) in S phase and in (**C**) in G2 phase.

Supplementary Table 1: Distribution of normal and CS primary fibroblasts into cell cycle phases.

	G1 phase (%)	S phase (%)	G2 phase (%)
N2RO	90	3,4	6,6
CS6PV	91	3,6	5,4
CS20PV	90	3,7	6,3

Normal: N2RO; CS-A: CS6PV; CS-B: CS20PV.
